# Levoketoconazole in the Treatment of Patients With Cushing’s Syndrome and Diabetes Mellitus: Results From the SONICS Phase 3 Study

**DOI:** 10.3389/fendo.2021.595894

**Published:** 2021-04-07

**Authors:** Rosario Pivonello, Atanaska Elenkova, Maria Fleseriu, Richard A. Feelders, Przemyslaw Witek, Yona Greenman, Eliza B. Geer, Paola Perotti, Leonard Saiegh, Fredric Cohen, Giorgio Arnaldi

**Affiliations:** ^1^Dipartimento di Medicina Clinica e Chirurgia, Sezione di Endocrinologia, Università Federico II di Napoli, Naples, Italy; ^2^Department of Endocrinology, Medical University-Sofia, Sofia, Bulgaria; ^3^Department of Medicine, Division of Endocrinology, Diabetes and Clinical Nutrition, and Department of Neurological Surgery, Oregon Health and Science University, Portland, OR, United States; ^4^Department of Medicine, Division of Endocrinology, Erasmus University Medical Center, Rotterdam, Netherlands; ^5^Department of Internal Medicine, Endocrinology and Diabetes, Medical University of Warsaw, Warsaw, Poland; ^6^Institute of Endocrinology and Metabolism, Tel Aviv-Sourasky Medical Center and Sackler Faculty of Medicine, Tel Aviv University, Tel Aviv, Israel; ^7^Pituitary and Skull Base Tumor Center, Memorial Sloan Kettering Cancer Center, New York, NY, United States; ^8^Department of Clinical and Biological Science, University of Turin, Orbassano, Italy; ^9^Department of Endocrinology, Bnai-Zion Medical Center, Haifa, Israel; ^10^Strongbridge Biopharma, Trevose, PA, United States; ^11^Division of Endocrinology, Polytechnic University of Marche Region, Ancona, Italy

**Keywords:** Cushing’s syndrome, Cushing’s disease, levoketoconazole, hypercortisolism, diabetes mellitus

## Abstract

**Background:**

Cushing’s syndrome (CS) is associated with numerous comorbidities, including diabetes mellitus (DM). Levoketoconazole, an orally administered ketoconazole stereoisomer, is in clinical trials for the treatment of CS.

**Methods:**

SONICS, a prospective, open-label, phase 3 study in adults with confirmed CS and mean 24-h urinary free cortisol (mUFC) ≥1.5× ULN, included dose-titration, 6-month maintenance, and 6-month extension phases. This subanalysis evaluated the efficacy of levoketoconazole in patients with DM (n = 28) or without DM (n = 49) who entered the maintenance phase. Safety was evaluated in the overall population (N = 94) during the dose-titration and maintenance phases.

**Results:**

Normalization of mUFC at the end of maintenance phase (EoM), without a dose increase during maintenance (SONICS primary endpoint) was observed in 46% of patients with DM (95% CI, 28 to 66%; *P* = 0.0006 *vs* null hypothesis of ≤20%) and 33% of patients without DM (95% CI, 20 to 48%; *P* = 0.0209). At EoM, mean HbA1c decreased from 6.9% at baseline to 6.2% in patients with DM and from 5.5 to 5.3% in patients without DM. Mean fasting blood glucose decreased from 6.85 mmol/L (123.4 mg/dl) to 5.82 mmol/L (104.9 mg/dl) and from 5.11 mmol/L (92.1 mg/dl) to 4.66 mmol/L (84.0 mg/dl) in patients with and without DM, respectively. Adverse events that were more common in patients with DM included nausea (58.3%), vomiting (19.4%), and urinary tract infection (16.7%); none prompted study drug withdrawal.

**Conclusions:**

Treatment with levoketoconazole led to sustained normalization of mUFC and improvement in glycemic control that was more pronounced in patients with DM.

**Clinical Trial Registration:**

(ClinicalTrials.gov), NCT01838551.

## Introduction

Cushing’s syndrome (CS) is characterized by chronic overproduction of cortisol and is associated with numerous comorbidities, one of which is diabetes mellitus (DM) (1–5). Diabetes mellitus has been diagnosed in 11 to 47% of patients with CS (6), with a higher prevalence compared to body mass index (BMI)-matched controls (7). The prevalence of DM may be higher in ectopic CS (74%) compared with pituitary-dependent CS or Cushing’s disease (33%) and adrenal CS (34%) (8). In addition, the prevalence of DM has been found to correlate with the duration and severity of hypercortisolism (9, 10). In patients with CS, DM is primarily the result of insulin resistance and impaired insulin secretion caused by hypercortisolism (11, 12), although various risk factors (age, genetic predisposition, lifestyle) also contribute (13, 14). In addition to causing pancreatic *β*-cell dysfunction, glucocorticoid excess produces anti-insulin effects in the liver (increased glucose production, inability of insulin to inhibit gluconeogenesis and glycogenolysis), skeletal muscle (decreased glucose uptake), and adipose tissue (increased visceral fat mass, inability of insulin to restrain lipolysis) (15–17). DM contributes to the excess mortality associated with CS, primarily due to cardiovascular disease (18, 19).

Medical therapy for patients with CS is indicated for persistent or recurrent disease after surgery, when surgery is not feasible or is declined by the patient, as preoperative treatment, and as a bridging therapy while awaiting the effects of radiosurgery (1, 3, 20, 21). Medical therapies for CS vary in their site of action and include pituitary-targeted agents, adrenal steroidogenesis inhibitors, and a glucocorticoid receptor antagonist (3, 22, 23). Levoketoconazole, an orally administered stereoisomer of racemic ketoconazole, inhibits key cytochrome P450 enzymes involved in multiple steps of steroidogenesis (24, 25) and is in ongoing clinical trials for the treatment of CS (1, 20, 26). Levoketoconazole was initially investigated as a potential treatment for DM; in small randomized, double-blind, placebo-controlled studies in patients with type 2 DM without CS, levoketoconazole reduced glycated hemoglobin (HbA1c) and fasting plasma glucose levels (27, 28).

Management of diabetes in patients with CS must concurrently address both hypercortisolism and hyperglycemia (15). In some patients, normalization of cortisol levels is sufficient to normalize glucose metabolism (29). Postsurgical hypopituitarism, a complication of pituitary surgery, may also lead to altered glucose metabolism (29). Sometimes, however, despite improvements in glycemic control obtained from surgery and/or medical therapy for CS, there is a need for continued antidiabetic therapy (15). In these patients, management typically follows the general standard of care for type 2 DM (15, 30).

The Study of levOketocoNazole In Cushing’s Syndrome (SONICS) was a prospective, open-label, phase 3 study that comprised a dose-titration phase (to establish a therapeutic dose), a 6-month maintenance phase, and a 6-month extended evaluation phase. The main results from SONICS through the end of the 6-month maintenance phase demonstrated that oral levoketoconazole treatment (150–600 mg twice daily) in adults with CS led to sustained reductions in mean 24-h urinary free cortisol (mUFC) levels, mean improvements in biomarkers of cardiovascular risk [fasting blood glucose, HbA1c, low-density lipoprotein (LDL)-cholesterol, body weight], and mean improvements in clinical signs of CS (acne, hirsutism in women, peripheral edema) (31).

Medical agents used in the treatment for CS may have direct effects on glucose metabolism that are not related to cortisol levels (11, 15). Ketoconazole, cabergoline, and osilodrostat may have positive effects on glucose metabolism, while pasireotide, especially in patients with diabetes, has been established to have negative effects (29, 32). Therefore, it is important to evaluate new medications for CS in the subpopulation of patients with diabetes. This *post hoc* analysis aimed to evaluate the efficacy and safety of levoketoconazole in the subset of SONICS participants with type 2 DM.

## Methods

### Study Design and Patients

The study methodology is described in detail elsewhere (31). Briefly, SONICS was a phase 3, multicenter, single-arm, non-randomized, open-label study of levoketoconazole that enrolled adults with a confirmed diagnosis of CS and mUFC levels ≥1.5× the upper limit of normal (ULN). All patients provided written informed consent to participate, and the study protocol was approved by an institutional review board or independent ethics committee at each center. During a dose-titration phase, levoketoconazole dose was adjusted (from 150 mg twice daily to a maximum of 600 mg twice daily) with a goal of achieving mUFC normalization with adequate tolerability. A therapeutic dose was determined based on either (a) reaching mUFC ≤ULN or (b) reaching levoketoconazole dose of 600 mg twice daily or a maximal tolerated dose with clinically meaningful partial cortisol response in the opinion of the investigator. Patients for whom a therapeutic dose was identified were eligible to continue into a 6-month maintenance phase, during which the established levoketoconazole dose was to remain unchanged, unless adjustment was needed to maintain control of hypercortisolism or address safety/tolerability issues. Certain concomitant medications were prohibited during the study [*e.g.*, strong cytochrome P450 3A4 inducers or inhibitors (contraindicated for use with ketoconazole), weight-loss medications, proton pump inhibitors]; antihyperglycemic, antihypertensive, and selected cholesterol-lowering medications were permitted but were to remain unchanged during the study unless necessary for managing the respective conditions.

The diagnosis of DM was recorded at study entry according to the judgments of the principal investigators based on medical history, current medications, and laboratory tests. Patients with poorly controlled diabetes (as evidenced by repeated hospitalizations for hyperglycemia or other serious complications of diabetes during the previous 12 months) were excluded. Patients diagnosed with DM at study entry were predefined as a subgroup of interest. Results through the end of the maintenance phase (EoM), which is the period predefined to include the primary and secondary efficacy outcomes, are summarized in the present analyses.

### Outcomes

The primary efficacy outcome of the main study was the percentage of patients demonstrating normalization of mUFC at the completion of 6 months of therapy in the maintenance phase without an increase in dose at any time during maintenance (*i.e.*, mUFC responders). Patients were considered non-responders if they did not have an mUFC value at EoM for any reason and/or had an increase in dose (transient or permanent) during maintenance. Patients with a history of radiation who exhibited no rebound increase in mUFC following brief withdrawal of levoketoconazole immediately after EoM were also considered non-responders. The main study definitions of response and non-response were carried over to the current exploratory analyses, which segregated the population according to DM status at study entry.

Key predefined secondary outcomes in the main study included measures of glycemic control [fasting blood glucose (change from baseline to Day 1 and monthly), HbA1c (change from baseline to Day 1 and months 3 and 6)] and other biomarkers of cardiovascular risk [change from baseline to EoM in lipid profile, blood pressure, C-reactive protein (CRP)]. Changes from baseline to EoM in concomitant medications (particularly antihyperglycemic, antilipidemic, and antihypertensive medications) were recorded. Safety assessments included adverse event (AE) reports, ECGs, and laboratory tests. Adverse events of special interest were liver enzyme elevations or other signs/symptoms of hepatic dysfunction, QTc interval prolongation, and adrenal insufficiency (which was identified based on multiple indicators, including clinical signs and symptoms, blood pressure measurements, and serum cortisol levels).

### Statistical Analysis

The intent-to-treat (ITT) population consisted of all patients who received at least one dose of levoketoconazole (N = 94). In these exploratory analyses, the proportion of mUFC responders at EoM was estimated for patients in the ITT population after first segregating the population according to DM status (with *vs* without DM) at study entry. mUFC response proportions were estimated *via* a generalized linear model with repeated measurements for nominal visit and patient as a random effect, with adjustments for baseline covariates: region (US or non-US), diagnosis of hypertension, age, sex, CS duration, prior CS medication, and prior radiation. The null hypothesis was that the proportion of responders at EoM was ≤20%. The proportions of responders among those entering maintenance phase (N = 77) and those completing maintenance phase (N = 61) were considered supportive to the ITT analysis. The proportions of responders for these two populations were calculated separately for the subgroups with and without DM using binomial proportion with corresponding 95% Clopper–Pearson confidence interval (CI). *P* values represent a continuity-corrected asymptotic test comparing the Month 6 responder proportions against *post hoc*–defined null hypotheses that the proportion of responders in each DM status subgroup was ≤20%.

Patients in the ITT population who entered the maintenance phase (N = 77) constitute the maintenance population; this population was used for the analyses of secondary efficacy endpoints. Changes from baseline to EoM in comorbidity biomarkers were estimated separately for patients with and without DM as exploratory analyses. Statistical significance within each subgroup was evaluated using paired t-tests (*vs* the null hypothesis of no change); *P* values were not adjusted for multiplicity. Correlations between baseline values, actual values, or changes in mUFC or body weight at Months 3 and 6 and baseline values or changes in HbA1c or fasting glucose at Months 3 and 6 were assessed using multiple regression. Safety was evaluated in the ITT population during the dose-titration and maintenance phases.

## Results

### Patients

**Figure 1:**
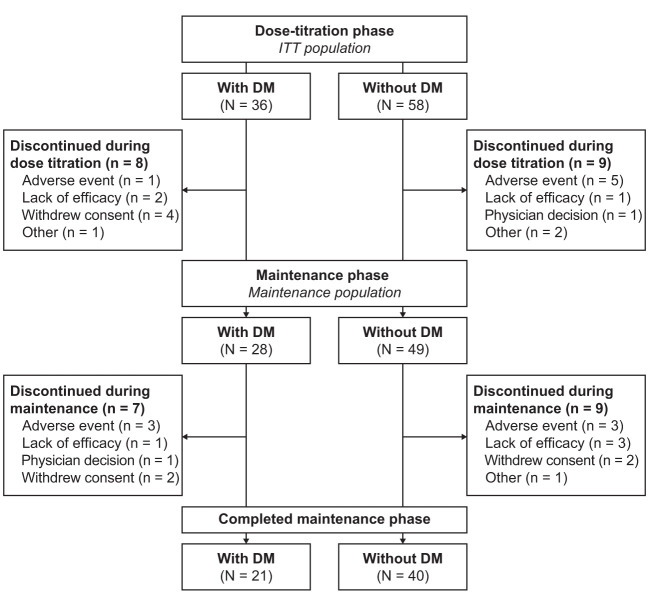
Patient disposition. ITT population included all patients who received ≥1 dose of study medication. Maintenance population consisted of all patients who entered the maintenance phase and received ≥1 dose of study medication during this phase. DM, diabetes mellitus; ITT, intent-to-treat.

The ITT population consisted of 94 patients, of whom 36 (38.3%) had DM (**Figure 1**). Twenty-eight of 36 patients (77.8%) with DM and 49 of 58 patients (84.5%) without DM continued into the maintenance phase (maintenance population); the maintenance phase completion rate was 58.3 and 69.0%, respectively. Baseline characteristics of patients with and without DM are summarized in **Table 1** and medications that were continued during the study in the subgroups are listed in the **Supplementary Table**. In the maintenance population, mean daily levoketoconazole dose during the maintenance phase was 562.7 mg [median (range): 448.7 mg (219.4–1164.0 mg)] in patients with DM and 603.3 mg [median (range): 576.4 mg (256.3–1182.8 mg)] in patients without DM.

**Table 1:** Patient demographics and baseline characteristics (ITT population).

Characteristic	Patients with DM (N = 36)	Patients without DM (N = 58)
Age, y, mean (SD)	48.3 (12.2)	40.8 (13.5)
Female, n (%)	33 (91.7)	44 (75.9)
Race, n (%)		
White	34 (94.4)	56 (96.6)
Black	0 (0)	1 (1.7)
Other	0 (0)	1 (1.7)
Unknown	2 (5.6)	0 (0)
Body mass index, kg/m^2^, mean (SD)	34.0 (8.4)	28.8 (7.5)
CS etiology, n (%)		
Cushing’s disease	29 (80.6)	51 (87.9)
Ectopic ACTH secretion	1 (2.8)	0 (0)
Adrenal dependent	4 (11.1)	4 (6.9)
Unknown	2 (5.6)	3 (5.2)
Diagnosis of hypertension, n (%)	32 (88.9)	35 (60.3)
Diagnosis of hypercholesterolemia, n (%)	21 (58.3)	13 (22.4)
Baseline mUFC, nmol/24 h		
Mean (SD)	579.6 (709.0)*^a^*	725.2 (763.4)
Median (range)	365.1 (162.0–4168.0)	420.3 (209.7–3817.0)
Baseline mUFC, mcg/24 h		
Mean (SD)	210.0 (256.9)*^a^*	262.7 (276.6)
Median (range)	132.3 (58.7–1510.1)	152.3 (76.0–1383.0)

a Baseline mUFC based on 34 patients.

ACTH, adrenocorticotropic hormone; CS, Cushing’s syndrome; DM, diabetes mellitus; ITT, intent-to-treat; mUFC, mean 24-hour urinary free cortisol; SD, standard deviation.

### Efficacy

In the ITT population, mean mUFC at baseline was 210.0 mcg/24 h (median: 132.3 mcg/24 h) in patients with DM and 262.7 mcg/24 h (median: 152.3 mcg/24 h) in patients without DM (approximately 4× to 5× the ULN value of 50 mcg/24 h). Normalization of mUFC at EoM without a dose increase during the maintenance phase (the study primary endpoint using a generalized linear model) was observed in 30% of patients in the overall ITT population (95% CI, 21 to 40%; one-sided *P* = 0.015), as previously reported (31). In the ITT population, mUFC normalization at EoM was observed in 13 (36%) of 36 patients with DM [least-squares (LS) mean estimate of 34% (95% CI, 19 to 53%); *P* = 0.035] and 16 (28%) of 58 patients without DM [LS mean estimate of 25% (95% CI, 15 to 39%); *P* = 0.196]. Exclusion of four patients who had baseline HbA1c ≥6.5% in the group without DM did not change the inference regarding mUFC normalization for this subgroup.

In the maintenance population, mUFC normalization at EoM was observed in 29 (38%) of 77 patients overall (95% CI, 27 to 49%; *P* < 0.0001), 13 (46%) of 28 patients with DM (95% CI, 28 to 66%; *P* = 0.0006), and 16 (33%) of 49 patients without DM (95% CI, 20 to 48%; *P* = 0.0209). Among patients completing the maintenance phase, normalization of mUFC was seen in 29 (48%) of 61 patients overall (95% CI, 35 to 61%; *P* < 0.0001), 13 (62%) of 21 patients with DM (95% CI, 38 to 82%; *P* < 0.0001), and 16 (40%) of 40 patients without DM (95% CI, 25 to 57%; *P* = 0.0015).

In the statistical model assessing mUFC normalization, diabetes was not a statistically significant factor predicting mUFC response in the overall ITT population [odds ratio (OR), 1.25; 95% CI, 0.53 to 2.96; *P* = 0.6058], maintenance population (OR, 1.38; 95% CI, 0.52 to 3.67; *P* = 0.5114), or among those completing the maintenance phase (OR, 2.45; 95% CI, 0.83 to 7.24; *P* = 0.1038).

**Figure 2:**
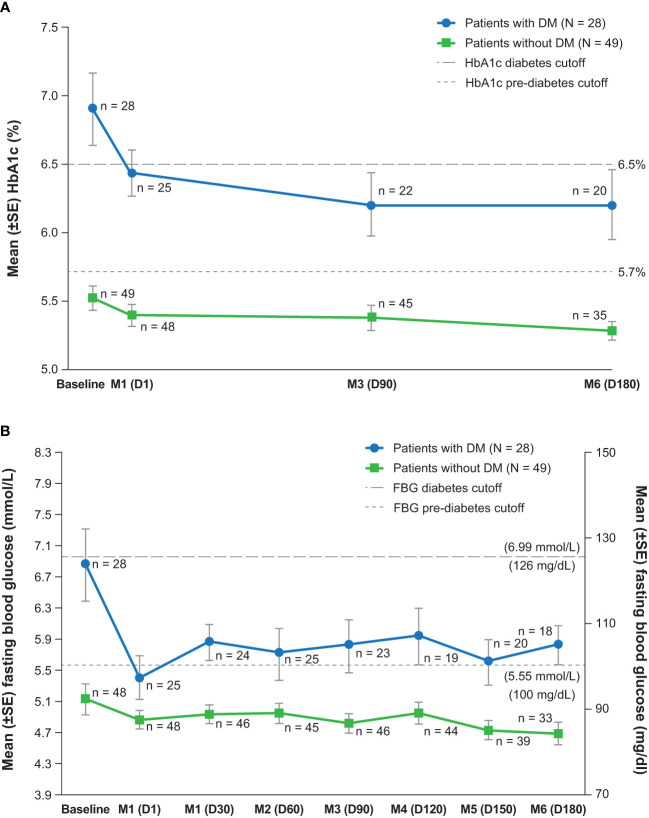
Measures of glycemic control over time, **(A)** hemoglobin A1c and **(B)** fasting blood glucose (maintenance population). D, day; DM, diabetes mellitus; FBG, fasting blood glucose; HbA1c, hemoglobin A1c; M, month; SE, standard error.

At baseline, HbA1c and fasting glucose levels were each linearly associated with the DM status at study entry (binary) but not with mUFC concentration or body weight. In patients with and without DM, significant mean improvements from baseline to EoM were observed in HbA1c and fasting blood glucose, with relatively larger reductions in those with DM (**Figure 2**). In patients with DM and HbA1c ≥6.5%, shift data showed improvement in HbA1c status in six of 13 patients (46%) and worsening in none (**Table 2**). Similarly, fasting blood glucose shifts indicated improvement to <6.1 mmol/L (110 mg/dl) in five of 10 (50%) DM patients with baseline fasting glucose >6.9 mmol/L (125 mg/dl). In patients without DM, improvement in HbA1c from ≥6.5% category was seen in two of three (67%) patients, and the one patient with fasting blood glucose >6.9 mmol showed improvement; instances of worsening were rare.

**Table 2:** Shift from baseline to Month 6 (or last assessed visit in the maintenance phase) in markers of glycemic control (maintenance population).

Patients with DM (N = 28)			Patients without DM (N = 49)		
Baseline category	End of maintenance phase category		Baseline category	End of maintenance phase category	
Hemoglobin A1c					
<5.7% (<38.8 mmol/mol) (n = 5)	<5.7%	4 (80%)	<5.7% (<38.8 mmol/mol)	<5.7%	34 (100%)
	5.7–<6.5%	1 (20%)	(n = 34)	5.7–<6.5%	0
	6.5–<8%	0		6.5–<8%	0
	≥8%	0		≥8%	0
5.7–<6.5% (38.8–<47.5 mmol/mol) (n = 9)	<5.7%	3 (33%)	5.7–<6.5% (38.8–<47.5 mmol/mol)	<5.7%	7 (58%)
	5.7–<6.5%	6 (67%)	(n = 12)	5.7–<6.5%	5 (42%)
	6.5–<8%	0		6.5–<8%	0
	≥8%	0		≥8%	0
6.5–<8% (47.5–<63.9 mmol/mol) (n = 6)	<5.7%	1 (17%)	6.5–<8% (47.5–<63.9 mmol/mol)	<5.7%	1 (33%)
	5.7–<6.5%	1 (17%)	(n = 3)	5.7–<6.5%	1 (33%)
	6.5–<8%	4 (67%)		6.5–<8%	0
	≥8%	0		≥8%	1 (33%)
≥8% (≥63.9 mmol/mol) (n = 7)	<5.7%	1 (14%)	≥8% (≥63.9 mmol/mol)	<5.7%	0
	5.7–<6.5%	2 (29%)	(n = 0)	5.7–<6.5%	0
	6.5–<8%	1 (14%)		6.5–<8%	0
	≥8%	3 (43%)		≥8%	0
Fasting Blood Glucose					
<6.1 mmol/L (<110 mg/dl) (n = 13)	<6.1 mmol/L	13 (10%)	<6.1 mmol/L (<110 mg/dl)	<6.1 mmol/L	41 (98%)
	6.1–6.9 mmol/L	0	(n = 42)	6.1–6.9 mmol/L	1 (2%)
	>6.9 mmol/L	0		>6.9 mmol/L	0
6.1–6.9 mmol/L (110–125 mg/dl) (n = 4)	<6.1 mmol/L	2 (50%)	6.1–6.9 mmol/L (110–125 mg/dl)	<6.1 mmol/L	4 (80%)
	6.1–6.9 mmol/L	1 (25%)	(n = 5)	6.1–6.9 mmol/L	1 (20%)
	>6.9 mmol/L	1 (25%)		>6.9 mmol/L	0
>6.9 mmol/L (>125 mg/dl) (n = 10)	<6.1 mmol/L	5 (50%)	>6.9 mmol/L (>125 mg/dl)	<6.1 mmol/L	1 (100%)
	6.1–6.9 mmol/L	3 (30%)	(n = 1)	6.1–6.9 mmol/L	0
	>6.9 mmol/L	2 (20%)		>6.9 mmol/L	0

DM, diabetes mellitus.

Linear correlates of changes in HbA1c and fasting glucose at Months 3 and 6 were explored using multiple linear regression. Baseline values of HbA1c and fasting glucose were correlated with changes in their respective outcomes at both Months 3 and 6. Body weight at Month 3 and its change to Month 3 each correlated with change in HbA1c at Month 3; they were not correlated with changes in fasting glucose at either time point. Mean UFC concentration, examined as a continuous variable, was not linearly correlated with either change in HbA1c or fasting glucose measured at either time point. However, mUFC examined categorically according to normalization state was linearly related to change in HbA1c at Month 3 only.

Owing to a recently discovered potential for drug-drug interaction between levoketoconazole and metformin that can lead to increased metformin plasma exposure (data on file), we qualitatively examined each case in which a patient with diagnosed diabetes used metformin on or before Month 6 (n = 15). In six of 15 (40%) patients, no improvement in HbA1c at Month 3 or Month 6 was noted. In the remaining nine (60%) patients, improvements in HbA1c were accompanied by improvements in mUFC and body weight and/or by changes in doses of antihyperglycemic medications.

Nominally significant mean improvements were seen in both patient subgroups for other key secondary endpoints, including reductions in total cholesterol, LDL-cholesterol, body weight, and BMI (**Table 3**). The mean reduction in waist circumference in patients with DM was nominally significant. There was a nominally significant mean decrease in high-density lipoprotein (HDL)-cholesterol in patients without DM only; a relatively smaller mean decrease in HDL-cholesterol was observed in patients with DM. Mean changes from baseline to Month 6 in systolic and diastolic blood pressure, triglycerides, and CRP were relatively small in both subgroups and not nominally significant. The directions of mean changes for these cardiovascular biomarkers varied between subgroups. As a notable example, mean triglycerides concentration increased (modestly) at Month 6 in the DM subgroup and did not change in the subgroup without DM.

**Table 3:** Change in key secondary endpoints from baseline to end of the maintenance phase (maintenance population).

Outcome measure	Patients with DM (N = 28)			Patients without DM (N = 49)			
	Baseline, mean (SD)	EoM, mean (SD)	*P* value*^a^*	Baseline, mean (SD)		EoM, mean (SD)	*P* value*^a^*
Fasting blood glucose, mmol/L mg/dl	6.85 (2.45) 123.4 (44.2) n = 28	5.82 (1.08) 104.9 (19.4) n = 18	0.046	5.11 (1.27) 92.1 (22.8) n = 48		4.66 (0.70) 84.0 (12.5) n = 33	0.044
Hemoglobin A1c, %	6.9 (1.4) n = 28	6.2 (1.1) n = 20	0.031	5.5 (0.5) n = 49		5.3 (0.4) n = 35	0.003
Total cholesterol, mmol/L mg/dl	5.45 (1.79) 210.4 (69.1) n = 27	4.62 (0.93) 178.2 (35.8) n = 20	0.004	5.76 (1.06) 222.4 (40.9) n = 48		4.63 (0.88) 178.9 (33.9) n = 34	<0.0001
LDL-cholesterol, mmol/L mg/dl	3.08 (1.39) 119.0 (53.7) n = 27	2.33 (0.89) 89.9 (34.5) n = 20	0.002	3.42 (1.04) 132.0 (40.3) n = 48		2.50 (0.71) 96.4 (27.4) n = 34	<0.0001
HDL-cholesterol, mmol/L mg/dl	1.48 (0.34) 57.2 (12.9) n = 27	1.40 (0.34) 54.0 (13.2) n = 20	0.107	1.72 (0.49) 66.4 (18.8) n = 48		1.52 (0.40) 58.7 (15.6) n = 34	0.001
Body weight, kg	86.6 (20.5) n = 28	81.3 (21.5) n = 20	<0.0001	79.5 (23.4) n = 49		76.3 (24.8) n = 34	0.004
BMI, kg/m^2^	33.5 (6.7) n = 28	31.2 (7.6) n = 20	0.0001	27.8 (6.4) n = 48		27.1 (7.7) n = 34	0.002
Abdominal girth, cm	111.8 (15.4) n = 20	99.7 (19.9) n = 17	0.038	100.9 (17.7) n = 28		95.2 (17.7) n = 22	0.268
Systolic blood pressure, mmHg	134.4 (18.1) n = 28	136.3 (21.3) n = 20	0.497	133.3 (14.4) n = 49		131.3 (16.2) n = 34	0.519
Diastolic blood pressure, mmHg	79.6 (11.2) n = 28	82.8 (11.6) n = 20	0.279	85.8 (13.3) n = 49		82.6 (11.7) n = 34	0.146
Triglycerides, mmol/L mg/dl	2.01 (1.64) 178.1 (144.9) n = 27	2.12 (1.56) 187.4 (137.8) n = 20	0.398	1.35 (0.69) 119.6 (61.2) n = 48		1.34 (0.73) 118.7 (64.6) n = 34	0.520
C-reactive protein, mg/L	4.9 (3.5) n = 27	4.7 (4.3) n = 19	0.640	3.4 (5.0) n = 47		3.8 (5.5) n = 32	0.572

a P values are from paired t tests and are considered descriptive.

BMI, body mass index; DM, diabetes mellitus; EoM, end of maintenance; HDL, high-density lipoprotein; LDL, low-density lipoprotein; SD, standard deviation.

Changes in concomitant medications from baseline (*i.e.*, before the first dose of levoketoconazole during the dose-titration phase) to the maintenance phase were evaluated as possible explanations for the improvements in markers of glycemic control and lipid parameters observed. There was no change in the net use of antidiabetic or antihypertensive medications among patients with DM. Concomitant antiglycemic medications used in this study were metformin, insulin and analogs, gliclazide, liraglutide, and sitagliptin. Exclusion of 10 (35.7%) patients with DM with any meaningful change in antidiabetic medication use did not affect inferences regarding mean changes in HbA1c and fasting glucose levels in this subgroup. Three patients with DM and three patients without DM started taking cholesterol-lowering medication during maintenance therapy (**Table 4**); removal of these six patients from the analyses did not have a meaningful effect on mean cholesterol changes in the overall population (data not shown).

**Table 4:** Changes from baseline in concomitant medication use (maintenance population; patients with DM, N = 28).*^a^*

Type of medication, n (%)	Patients taking medications before the start of levoketoconazole						Medication started after baseline
	Total	Started new and significant medication	Dose increased or restarted after gap	Dose decreased	No change from baseline	Stopped taking medication	
Antidiabetic	23 (82.1)*^b^*	2 (7.1)	1 (3.6)	3 (10.7)	13 (46.4)	3 (10.7)	2 (7.1)
Cholesterol-lowering	8 (28.6)	0	0	1 (3.6)	7 (25.0)	0	3 (10.7)
Antihypertensive	21 (75.0)*^b^*	2 (7.1)	3 (10.7)	3 (10.7)	11 (39.3)	1 (3.6)	1 (3.6)

^a^Worst (or most clinically significant) change during the maintenance phase.

^b^One patient had a clinically insignificant change (same dose of a different formulation or pharmacological equivalent dose of a different drug).

DM, diabetes mellitus.

### Safety

The overall incidence of treatment-emergent AEs in the ITT population was 97.2% in patients with DM and 98.3% in patients without DM. The most common AEs in patients with DM were nausea, vomiting, and urinary tract infection, whereas headache, peripheral edema, and hypertension were the most common among patients without DM (**Table 5**). Nausea, vomiting, and urinary tract infection events did not prompt study withdrawal in either subgroup; headache prompted one study withdrawal (patient without DM). Adrenal insufficiency was reported as an AE in one patient (2.8%) with DM and two patients (3.4%) without DM. The proportion of patients who permanently discontinued study medication due to an AE was 11.1% of those with DM and 13.8% of those without DM.

**Table 5:** Adverse events during the dose titration and maintenance phases (ITT population).

Adverse events, n (%)	Patients with DM (N = 36)	Patients without DM (N = 58)
Patients with at least 1 TEAE	35 (97.2)	57 (98.3)
Most common TEAEs (incidence ≥15% in either group)		
Nausea	21 (58.3)	9 (15.5)
Vomiting	7 (19.4)	3 (5.2)
Urinary tract infection	6 (16.7)	5 (8.6)
Fatigue	5 (13.9)	10 (17.2)
Headache	5 (13.9)	21 (36.2)
Hypertension	5 (13.9)	11 (19.0)
Peripheral edema	5 (13.9)	13 (22.4)
Diarrhea	4 (11.1)	10 (17.2)
Alopecia	0 (0)	9 (15.5)
ALT increased	4 (11.1)	10 (17.2)
AST increased	2 (5.6)	9 (15.5)
GGT increased	3 (8.3)	9 (15.5)
Patients with TEAEs probably or definitely related to study drug, n (%)	15 (41.7)	25 (43.1)
Patients discontinued due to TEAEs, n (%)	4 (11.1)	8 (13.8)
Patients with treatment-emergent SAEs, n (%)	7 (19.4)	7 (12.1)
Patients with treatment-emergent SAEs probably or definitely related to study drug, n (%)	3 (8.3)	1 (1.7)
Patients with adrenal insufficiency, n (%)	1 (2.8)	2 (3.4)

ALT, alanine aminotransferase; AST, aspartate aminotransferase; DM, diabetes mellitus; GGT, gamma-glutamyl transferase; ITT, intent-to-treat; SAE, serious adverse event; TEAE, treatment-emergent adverse event.

Laboratory monitoring showed that 10 patients (two with DM, eight without DM) had at least one alanine aminotransferase (ALT) elevation of more than 3× ULN. Three patients without DM and no patients with DM were found to have at least one ALT elevation of more than 5× ULN. Four patients with DM and seven patients without DM had one or more gamma-glutamyl transferase elevation of more than 3× ULN. No patients with DM and four patients without DM had one or more aspartate aminotransferase elevation of more than 3× ULN. No patients in either subgroup had one or more elevation of more than 3× ULN for alkaline phosphatase and more than 2× ULN for total bilirubin.

Among patients with postbaseline QTc data (35 patients with DM and 53 patients without DM), an increase from baseline greater than 60 ms in the Fridericia-corrected QT value (QTcF) was observed in nine patients [three with DM (8.6%); six without DM (11.3%)] at any time prior to the end of maintenance. Two patients (one with and one without DM) had a QTcF interval of >500 ms.

## Discussion

Diabetes mellitus is a common complication of CS (6). These exploratory analyses of a prospective phase 3 trial evaluated the efficacy and safety of levoketoconazole, the cis-2S,4R enantiomer of ketoconazole, in patients with CS with and without DM diagnosed prior to or at study entry. Normalization of mUFC after 6 months of maintenance treatment without a dose increase in levoketoconazole occurred in 34% of patients with DM and 25% of patients without DM in the overall study population and in 46 and 33% of patients, respectively, in the maintenance population. Notably, the mUFC analysis employed in these exploratory analyses mirrored the main study’s primary analysis: both used a conservative approach in which patients who discontinued from the study before EoM, regardless of the reason, or who did not have adequate Month 6 mUFC data, were considered to be non-responders. This approach was chosen to provide assurance of therapeutic efficacy, given the lack of a placebo comparison, which was necessitated by practical and ethical considerations. The apparently relatively higher rate of mUFC normalization at EoM among patients with a diagnosis of DM compared with patients without a diagnosis of DM at study entry was unexpected *a priori* and has no obvious mechanistic explanation. As these are *post hoc* subgroup analyses and both subgroups are relatively small, any apparent difference in response rates between subgroups may reflect sampling variability rather than a true difference. Notably, the confidence intervals around the estimated odds ratios for diabetes as a predictor of mUFC response were wide and included 1.0. A second prospective study of levoketoconazole, the LOGICS study, is underway (33). LOGICS is, similarly, not designed to determine baseline predictors of mUFC response, including DM, definitively; however, it is expected to provide additional data.

Despite modest baseline hyperglycemia, significant mean improvements in glycemic control were observed in patients with DM who received maintenance treatment with levoketoconazole. The effects on the glucose profile were observed throughout the maintenance phase (**Figure 2**), which was preceded by a 2- to 21-week dose titration phase. Mean duration of levoketoconazole exposure in the dose titration phase in the overall ITT population was 99.9 days [median 91.5 days (range: 17–256 days)] and was similar for both subgroups. Significant, albeit smaller, mean improvements were also demonstrated in the subgroup without DM, indicating that reduction in glycemia was not exclusive to patients with diabetes. Importantly, improvement in glycemia was not explained by adjustments in antidiabetic medications, as there was effectively no net change in their use.

In patients with CS, reductions in cortisol are generally associated with improvements in glycemic control (9); furthermore, cortisol normalization has been observed to reverse DM in some cases (34–36). Thus, medical therapies that provide biochemical control of hypercortisolism would be expected to reduce hyperglycemia in patients with DM; however, medications that treat CS may also affect glucose homeostasis by other mechanisms (9, 11, 15). In the present study, the normalization of mUFC was associated with an improvement of diabetic status (observed during the first 3 months of treatment), perhaps mediated by the concurrent reduction of body weight. However, there were no consistently significant correlations between changes in mUFC or body weight and improvements in fasting blood glucose or HbA1c. As was the case for mUFC response, a larger population in each subgroup at 6 months of maintenance treatment would help to clarify this issue. Notably, some comorbidities of CS are not reversible in all patients, even when cortisol levels are reduced; for example, diabetes may persist in 30 to 60% of patients despite sustained biochemical remission (37), which might obscure relationships between cortisolemia and glycemic biomarkers.

While two drugs used to treat CS, mifepristone (38) and cabergoline (39), have been shown to reduce glycemia, this is, to our knowledge, the first prospective phase 3 study on an adrenal steroidogenesis inhibitor that demonstrates this effect. By contrast, the somatostatin receptor ligand pasireotide, despite reducing cortisol levels, also decreases insulin secretion, which may result in a net negative effect on glucose metabolism (9, 29). In studies of patients with CS receiving pasireotide, hyperglycemia and DM were frequently observed (40–44).

Significant mean improvements in other cardiovascular risk markers (*i.e.*, LDL-cholesterol, weight, BMI) were seen in both patient subgroups, with a significant, though relatively smaller, mean decrease in HDL-cholesterol level opposing these improvements in patients without DM. Decreases in total and LDL-cholesterol with levoketoconazole might have been expected, given the known effect of ketoconazole in reducing serum cholesterol possibly *via* decreased bile acid synthesis and cholesterol homeostasis (45, 46). It is reassuring that these beneficial effects appear to be at least as strong in patients with DM compared with those without DM. Mean serum triglycerides did not change significantly during treatment in either patient subgroup, which argues against triglycerides being solely responsible for the HDL-cholesterol decrease in patients without DM. Reduction in serum HDL-cholesterol also has been observed with the glucocorticoid receptor antagonist mifepristone (47), suggesting that this effect may be mediated by alterations in cortisol synthesis or action. Improvements in cardiovascular risk markers, like those observed in this study, are potentially of heightened relevance in the CS population, as excess mortality in CS is largely explained by cardiovascular-specific mortality (3, 6, 48). Weight loss, seen in both subgroups, as an independent factor could have additional importance for motivating patients with DM to remain adherent to chronic medical therapy (49, 50).

The tolerability of levoketoconazole was good in patients with and without DM, as evidenced by the low rate of discontinuation related to AEs across both patient groups. However, the incidence of specific AEs varied by subgroup, as patients with DM more frequently reported gastrointestinal-related symptoms including nausea and vomiting, and urinary tract infections. These AEs may be related to DM itself (*i.e.*, diabetic gastropathy, immune suppression, and/or intermittent glucosuria) or to concomitant medications used to treat diabetes, especially metformin, which was an antidiabetic drug commonly used in this study. In addition, steroid withdrawal is a possible cause of nausea and other gastrointestinal symptoms. The liver test abnormalities in both subgroups were mild to moderate in severity and were fully reversible by drug discontinuation, with no clinical sequelae in any patient.

Open-label design, absence of a control group, and the *post hoc* nature of the analyses are the main limitations of the study. Although subgroup analysis based on DM status at study entry was predefined, the study was not powered to observe significant differences in outcomes from baseline to EoM or between subgroups. The effects of diet, if any, were not assessed in these subgroups, as diet was not monitored formally; however, body weight and abdominal girth were assessed and have been reported here. These analyses are therefore considered exploratory and require validation from an independent study.

## Conclusion

Levoketoconazole normalized mean mUFC and improved glucose control after 6 months of maintenance therapy in the overall study population in SONICS. The current subgroup analyses characterized these effects in patients with and without DM, revealing a greater glycemic reduction in the DM subgroup and possibly a greater rate of mUFC normalization. Awareness of the effect of different drugs for CS on glycemic control is important, as this may affect drug choice and diabetes medication adjustment. The safety profile appears similar in the two subgroups, suggesting that no special precautions are required for patients with DM other than routine glucose and cardiovascular risk factor monitoring.

## Sonics Investigator List

Principal investigators (N = number of patients enrolled in the study): **Belgium:** Marie Bex (University Hospitals Leuven; N = 3); **Bulgaria:** Sabina Zacharieva (Acad. Ivan Penchev; N = 6); **Canada:** Ehud Ur (St. Pauls Hospital/Vancouver General Hospital; N = 1); **Czechia:** Vaclav Hana (Vseobecna fakultni nemocnice v Praze–III. Interni klinika VFN a 1. LF UK; N = 0); **Denmark:** Marianne Andersen (Odense Universitets Hospital; N = 0); Ulla Feldt-Rasmussen (Rigshospitalet, Copenhagen University Hospital; N = 3); Caroline Kistorp (Herlev Hospital, Research Unit; N = 0); Logstrup Poulsen (Aarhus University Hospital; N = 1); **France:** Thierry Brue (Hopital de la CONCEPTION Service d’Endocrinologie, Diabete et Maladies Metaboliques; N = 2); **Georgia:** David Metreveli (David Metreveli Medical Centre; N = 0); **Germany:** Georg Brabant (Med Clinic I-University of Lubeck; N = 1); Gunter Stalla (Max-Plack-Institute of Psychiatry; N = 0); **Hungary:** Laszlo Kovacs (MH-Egeszsegugyi Kozpont; N = 0); Miklos Toth (Semmelweis University; N = 0); **Israel:** Faiad Adawi (Ziv Medical Center; N = 0); Yona Greenman (Sourasky Medical Center; N = 4); Leonard Saiegh (Bnai Zion Medical Center Institute of Endocrinology and Metabolism; N = 3); Ilan Shimon (Institute of Endocrinology and Metabolism, Rabin Medical Center, Beilinson Campus; N = 2); **Italy:** Giorgio Arnaldi (Azienda Ospedaliera-Universitaria Ancona; N = 3); Salvatore Cannavo (UOC di Endocrinologia, Dipartimento di Medicina, AOU Policlinico G. Martino; N = 2); Maria Vittoria Davi (Policlinico GB Rossi; N =0); Diego Ferone (University of Genova, IRCCS AOU San Martino-IST; N = 0); Roberta Giordano (Azienda Ospedaliero-Universitaria Città della Salute e della Scienza di Torino; N = 0); Massimo Mannelli (Azienda Ospedaliero - Universitaria Careggi; N = 0); Francesca Pecori Giraldi (Istituto Auxologico Italiano; N = 3); Rosario Pivonello (University of Naples Federico II; N = 6); Alfredo Pontecorvi (Policlinico Universitario Agostino Gemelli; N = 1); Carla Scaroni (University of Padua; N = 2); Massimo Terzolo (SCDU Medicina Interna I, Universita di Torino; N = 3); Vincenzo Toscano (UOC Endocrinologia, Azienda Ospedaliera Sant’Andrea; N = 0); **Netherlands:** Nienke Biermasz (Leiden University Medical Center; N = 1); Richard Feelders (Polikliniek Endocrinologie, Erasmus MC; N = 4); **Poland:** Marek Bolanowski (Samodzielny Publiczny Szpital Kliniczny Nr 1; N = 1); Andrzej Lewinski (Instytut Centrum Zdrowia Matki Polki; N = 1); Beata Matyjaszek-Matuszek (Terpa Sp.z.o.o; N = 2); Marek Ruchala (Szpital Kliniczny im. Heliodora Swiecickiego; N = 0); Przemyslaw Witek (Outpatient Clinic: Reuma Centrum; N = 4); **Serbia:** Milica Medic-Stojanoska (Clinical Center of Vojvodina Clinic for Endocrinology; N = 0); Sandra Pekic-Djurdjevic (Clinical Center of Serbia; N = 1); **Spain:** Carmen Fajardo (Hospital Universidad de la Ribera; N = 1); Maria Angeles Galvez (Hospital Universitario Reina Sofia; N = 1); Susan Webb (Hospital de la Santa Creu i Sant Pau; N = 1); **Sweden:** Gudmundur Johannsson (Sahlgrenska University Hospital; N = 0); **Turkey:** Abdurrahman Comlekci (Dokuz Eylul University Medical Faculty; N = 0); Pinar Kadioglu (Istanbul University Medical Faculty; N = 1); Ertugrul Tasan (Bezmi Alem Vakif Universitesi Endokrinoloji Bolumu Adnan; N = 2); **UK:** Tara Kearney (Salford Royal NHS Foundation Trust; N = 0); David Ray (Manchester Royal Infirmary; N = 0); **USA:** Richard Auchus (University of Michigan Medical Center; N = 4); Beverly Biller (Massachusetts General Hospital; N = 1); Maria Fleseriu (Oregon Health and Science University; N = 5); Eliza Geer (Memorial Sloan Kettering Cancer Center; N = 2; Icahn School of Medicine at Mount Sinai; N = 2); Hans Ghayee (University of Florida; N = 0); Murray Gordon (Allegheny Neuroendocrinology Center; N = 2); Anthony Heaney (University of California, Los Angeles, School of Medicine; N = 3); Patricia Kapsner (University of New Mexico HSC; N = 1); Laurence Kennedy (Cleveland Clinic; N = 3); Roberto Salvatori (Johns Hopkins University; N = 5); Kevin Yuen (Swedish Hospital; N = 0).

## Data Availability Statement

The raw data supporting the conclusions of this article will be made available by the authors, without undue reservation.

## Ethics Statement

The studies involving human participants were reviewed and approved by several institutional review boards. The patients/participants provided their written informed consent to participate in this study.

## Author Contributions

RP enrolled patients in study; collected data; contributed to interpreting data, writing, and revising report; and approved final version. AE enrolled patients in study; collected data; contributed to interpreting data, writing, and revising report; and approved final version. MF contributed to design of the study; enrolled patients in study; collected data; contributed to interpreting data, writing, and revising report; and approved final version. RF enrolled patients in study; collected data; contributed to interpreting data, writing, and revising report; and approved final version. PW enrolled patients in study; collected data; contributed to interpreting data, writing, and revising report; and approved final version. YG enrolled patients in study; collected data; contributed to interpreting data, writing, and revising report; and approved final version. EG enrolled patients in study; collected data; contributed to interpreting data, writing, and revising report; and approved final version. PP enrolled patients in study; collected data; contributed to interpreting data, writing, and revising report; and approved final version. LS contributed to interpretation of data; critically reviewed and revised manuscript; and approved final version. FC contributed to interpretation of data; critically reviewed and revised manuscript; and approved final version. GA enrolled patients in study; collected data; contributed to interpreting data, writing, and revising report; and approved final version. All authors contributed to the article and approved the submitted version.

## Funding

The study was funded by Cortendo AB (a subsidiary of Strongbridge Biopharma) and funding for editorial assistance was provided by Strongbridge Biopharma.

## Conflict of Interest

The authors declare that this study was funded by Cortendo AB (a subsidiary of Strongbridge Biopharma). Strongbridge Biopharma was involved in the study design and implementation, data collection and analysis, and critical review of the report.

RP reports serving as the principal investigator of research grants to Federico II University from Corcept Therapeutics, Novartis, and Strongbridge Biopharma and receiving consulting honoraria from Novartis and Strongbridge Biopharma. AE reports serving as the principal investigator/sub-investigator of clinical trials for Corcept Therapeutics and Novartis and receiving consulting honoraria from Novartis. MF reports serving as an investigator with research grants to OHSU for Novartis and Strongbridge and serving as an occasional consultant to Novartis and Strongbridge. RF reports receiving research grants from Novartis and serving on the speakers’ bureau for HRA Pharma and Novartis. PW reports receiving travel grants from Ipsen and Novartis and receiving personal fees as a clinical investigator from Ipsen, Novartis, Novo Nordisk, and Strongbridge Biopharma. YG reports serving as the principal investigator of research grants to Tel Aviv-Sourasky Medical Center from Chiasma, Novartis, and Strongbridge Biopharma and receiving lecture fees from Medison, Novartis, and Pfizer. EG reports serving as an investigator for research grants to MSKCC from Ionis, Novartis, and Strongbridge Biopharma and serving as an occasional consultant to Novartis and Strongbridge Biopharma. LS reports receiving lecture fees from Novartis and receiving travel grants from Medison and Novartis. FC is an employee of Strongbridge Biopharma. GA reports receiving lecture fees from Novartis and Otsuka and receiving consulting honoraria from HRA Pharma.

The remaining author declares that the research was conducted in the absence of any commercial or financial relationships that could be construed as a potential conflict of interest.
