# Supplementary material for: Levoketoconazole in the Treatment of Patients With Cushing’s Syndrome and Diabetes Mellitus: Results From the SONICS Phase 3 Study
Source: Front Endocrinol (Lausanne). 2021 Apr 7;12:595894. doi: 10.3389/fendo.2021.595894 (PMC8059833; doi:10.3389/fendo.2021.595894)
Supplement: Supplementary file 1 [file Table_1.docx]

| **Supplementary Table\| Most common continuing medications^a^ (ITT population)** | | |
| --- | --- | --- |
| **Medication class^b^, n (%)** | **Patients with DM**  **(N = 36)** | **Patients without DM**  **(N = 58)** |
| Any continuing medication | 36 (100.0) | 55 (94.8) |
| Most common continuing medications^c^ |  |  |
| Blood glucose lowering drugs (excluding insulin) | 25 (69.4) | 5 (8.6) |
| Vitamin A and D (including combinations) | 18 (50.0) | 24 (41.4) |
| Beta blocking agents | 14 (38.9) | 13 (22.4) |
| Lipid modifying agents (plain) | 13 (36.1) | 9 (15.5) |
| Insulin and analogues | 12 (33.3) | 0 |
| Thyroid preparations | 12 (33.3) | 17 (29.3) |
| Angiotensin II antagonists (plain) | 10 (27.8) | 10 (17.2) |
| Angiotensin-converting enzyme inhibitors (plain) | 9 (25.0) | 19 (32.8) |
| Antithrombotic agents | 8 (22.2) | 11 (19.0) |
| Selective calcium channel blockers with mainly vascular effects | 8 (22.2) | 9 (15.5) |
| Antacids | 7 (19.4) | 5 (8.6) |
| Anti-inflammatory and antirheumatic products (nonsteroids) | 7 (19.4) | 9 (15.5) |
| Calcium | 7 (19.4) | 16 (27.6) |
| Antidepressants | 6 (16.7) | 11 (19.0) |
| High-ceiling diuretics | 6 (16.7) | 4 (6.9) |
| Other analgesics and antipyretics | 6 (16.7) | 9 (15.5) |
| Potassium | 6 (16.7) | 8 (13.8) |
| Drugs affecting bone structure and mineralization | 5 (13.9) | 6 (10.3) |
| Low-ceiling diuretics, thiazides | 5 (13.9) | 8 (13.8) |
| Potassium-sparing agents | 5 (13.9) | 4 (6.9) |
| Antiadrenergic agents (peripherally acting) | 4 (11.1) | 3 (5.2) |
| Iron preparations | 4 (11.1) | 6 (10.3) |
| Multivitamins (combinations) | 4 (11.1) | 3 (5.2) |
| Opioids | 4 (11.1) | 1 (1.7) |
| Other mineral supplements | 3 (8.3) | 8 (13.8) |
| Vitamin B12 and folic acid | 2 (5.6) | 7 (12.1) |
| Unspecified herbal and traditional medicine | 2 (5.6) | 6 (10.3) |

^a^Medications that were started before the date of the first dose of levoketoconazole, stopped on or after the date of the first dose of the study drug.

^b^Classification based on WHO Drug September 2018 version.

^c^Medication used by ≥10% of the patients in either subgroup.

DM, diabetes mellitus; ITT, intent-to-treat.
